# Real-time algorithm for Poissonian noise reduction in low-dose fluoroscopy: performance evaluation

**DOI:** 10.1186/s12938-019-0713-7

**Published:** 2019-09-11

**Authors:** A. Sarno, E. Andreozzi, D. De Caro, G. Di Meo, A. G. M. Strollo, M. Cesarelli, P. Bifulco

**Affiliations:** 1 Università di Napoli, “Federico II”, dip. di Fisica “E. Pancini” & INFN sez. di Napoli, Via Cintia, 80126 Naples, Italy; 2 Department of Electrical Engineering and Information Technologies, Università di Napoli “Federico II”, Via Claudio, 21, 80125 Naples, Italy; 3 Istituti Clinici Scientifici Maugeri S.p.A.—Società Benefit, Via S. Maugeri, 4, 27100 Pavia, Italy

**Keywords:** Fluoroscopy, Quantum noise, Video denoising, Real-time processing, X-ray dose reduction, NVCA, VBM4D

## Abstract

**Background:**

Quantum noise intrinsically limits the quality of fluoroscopic images. The lower is the X-ray dose the higher is the noise. Fluoroscopy video processing can enhance image quality and allows further patient’s dose lowering. This study aims to assess the performances achieved by a Noise Variance Conditioned Average (NVCA) spatio-temporal filter for real-time denoising of fluoroscopic sequences. The filter is specifically designed for quantum noise suppression and edge preservation. It is an average filter that excludes neighborhood pixel values exceeding noise statistic limits, by means of a threshold which depends on the local noise standard deviation, to preserve the image spatial resolution. The performances were evaluated in terms of contrast-to-noise-ratio (CNR) increment, image blurring (full width of the half maximum of the line spread function) and computational time. The NVCA filter performances were compared to those achieved by simple moving average filters and the state-of-the-art video denoising block matching-4D (VBM4D) algorithm. The influence of the NVCA filter size and threshold on the final image quality was evaluated too.

**Results:**

For NVCA filter mask size of 5 × 5 × 5 pixels (the third dimension represents the temporal extent of the filter) and a threshold level equal to 2 times the local noise standard deviation, the NVCA filter achieved a 10% increase of the CNR with respect to the unfiltered sequence, while the VBM4D achieved a 14% increase. In the case of NVCA, the edge blurring did not depend on the speed of the moving objects; on the other hand, the spatial resolution worsened of about 2.2 times by doubling the objects speed with VBM4D. The NVCA mask size and the local noise-threshold level are critical for final image quality. The computational time of the NVCA filter was found to be just few percentages of that required for the VBM4D filter.

**Conclusions:**

The NVCA filter obtained a better image quality compared to simple moving average filters, and a lower but comparable quality when compared with the VBM4D filter. The NVCA filter showed to preserve edge sharpness, in particular in the case of moving objects (performing even better than VBM4D). The simplicity of the NVCA filter and its low computational burden make this filter suitable for real-time video processing and its hardware implementation is ready to be included in future fluoroscopy devices, offering further lowering of patient’s X-ray dose.

## Background

Fluoroscopy is a medical imaging modality able to provide continuous real-time X-ray screening of patient’s body parts (also highlighted by contrast agents), as well as various radiopaque surgical instruments, catheters, wire-guides, and prosthetic implants. Thus, it is an invaluable tool for interventional radiology procedures, such as orthopedic surgery, angioplasty, pacemaker and defibrillator implantation [1, 2], for diagnostic exams, such as investigations of gastrointestinal tract or blood vessels, for the assessments of joints and implanted prosthesis [3, 4] and image-guided radiotherapy [5]. Since these applications require extended and unpredictable exposure times, patient radiation dose must be kept acceptably low, resulting in a degradation of image quality. In fact, the limited number of X-ray photons per pixel produces a signal-dependent, Poisson-distributed noise, also known as “quantum noise” [6], which causes a significant decrease in the signal–noise ratio (SNR) and contrast–noise ratio (CNR). Quantum noise is by far the most dominant noise source in low-dose X-ray images [7–10] and cannot be avoided by improving sensors/detectors technology, since it is inherent to the image formation process.

The statistical characteristics of the Poisson distribution imply that the SNR is equal to the square root of the mean photon count $\lambda$ (${\text{SNR}} = \sqrt \lambda$). This means that the lower the dose, the worse the image quality. A better image quality could be only achieved by increasing the number of photons per pixel. Unfortunately, this cannot be actually pursued in fluoroscopy, since it would require an unacceptable increase of patient’s dose, as exposure time cannot be reduced or even limited. Thus, there is the need to improve fluoroscopic image quality by means of image processing techniques.

Nowadays, commercial fluoroscopic devices implement low-dose protocols by reducing the frame rate or performing simple temporal or spatial averages to allow real-time denoising (essential to support clinical interventional procedures). However, both temporal and spatial averaging tend to produce undesirable blurring effects, which undermine edges preservation and produce motion blur. Moreover, the averaging operation is fully effective only if the averaged samples are uncorrelated. Although this is practically always verified in the time domain, since the lag times of scintillators are about 1 ms [11] while the minimum frame interval is about 33 ms (i.e., 30 fps), it is not verified in the spatial domain, as it depends on the specific point spread function of the fluoroscopy device. Thus, temporal averaging is practically always implemented in commercial devices, while spatial averaging is not very common.

Spatio-temporal averaging acts as a simple low-pass filter; therefore, it reduces not only the noise spectral power, but also the high-frequency content of the useful signal (i.e., static and moving edges). This results in poor denoising performances and makes these approaches unsuitable for several applications, such as derivative-based techniques (e.g., image segmentation, object recognition, image registration), as they are particularly sensitive to noise and edge blurring [6, 10]. It is clear that more efficient denoising methods, which can achieve higher SNRs while reducing or avoiding these side effects, would be of help.

The literature offers many denoising algorithms, but their effectiveness strongly depends on the validity of the noise model [6, 12, 13]. Most of these methods assume the noise to be space-invariant, additive, white and Gaussian (AWGN), which is not the case in fluoroscopic images as noise is rather space-variant and Poisson distributed. However, some techniques aim to stabilize the Poisson’s noise variance, e.g., via the Anscombe transformation, to apply AWGN denoising techniques [14–16]. Unfortunately, the Anscombe transform suffers from different limitations. First of all, it cannot be exactly inverted, as the inverse transform introduces bias errors [14, 15]. Also, it can be directly applied only to Poisson distributions, resulting unsuitable for real fluoroscopic data, as their original Poisson statistics are usually modified by sensor non-linearities (e.g., clipping effects) and white compression operations commonly implemented in commercial fluoroscopic devices (e.g., log-mapping, gamma-correction) [17]. Inverting these non-linearities would require additional operations, resulting in a further increase of the computational burden. Moreover, these techniques do not allow an easy real-time implementation, rather being suitable for accurate post-processing. Other recent approaches mainly concentrate on edge enhancement [18, 19] and on recognition of curvilinear guide-wires [20] in fluoroscopy but they do not provide a global approach to noise reduction.

The Noise Variance Conditioned Average (NVCA) algorithm [17], which considers the specific signal-dependent, Poisson-distributed nature of the quantum noise, has proven to be more efficient than several algorithms in the denoising of X-ray images [21], while keeping the computational burden low enough to allow real-time hardware implementations [22, 23].

This study aims to evaluate the performance of the NVCA algorithm in terms of noise reduction and edge preservation for static and moving objects. In particular, it was tested using different phantoms and real fluoroscopy sequences where a small radiopaque needle is present. The NVCA algorithm was compared to the simple moving average filter and to the Video Block-Matching and 4D joint filtering (VBM4D) algorithm [24, 25] (after noise variance stabilization via generalized Anscombe transform [15]), which was assumed as the reference state-of-the-art video denoiser. The VBM4D is an improved version of the VBM3D which, in turn, is an extension for video processing of the famous BM3D, and still considered as one of the best methods for image denoising [26–29]. The BM3D extensions for video processing (such as VBM3D and VBM4D) are likewise widely regarded as the state-of-the-art for video denoising, and, indeed, they have been used as a reference for the performance assessment of new algorithms in many recent studies [30–32].

The “Results” section shows the performances of the denoising algorithms in terms of spatial resolution (evaluated as the full width at half maximum (FWHM) of the line spread function evaluated either across static or moving object), object visibility and computational time, as well as the effect of motion blur in filtered images. Also a global image quality index, the feature similarity index (FSIM) was evaluated. The “Discussion” and “Conclusion” sections summarize the issues addressed, the results obtained and the possible practical implications. The “Methods” section describes the noise model adopted for fluoroscopy images, the proposed denoising algorithm and the reference algorithms used for comparison, the phantoms and the images used to test the denoising performances and the parameters used to assess image quality.

## Results

### Spatio-temporal correlations and expected value–variance relationship

**Fig. 1:**
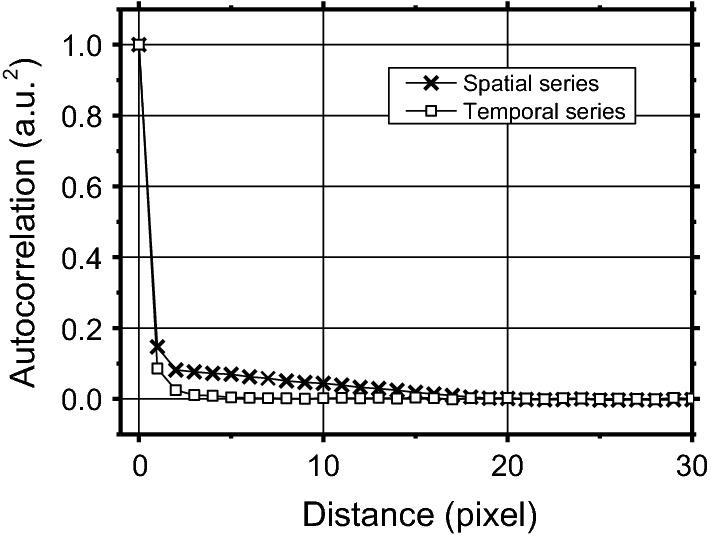
Average autocorrelation function evaluated in space (black crosses) and over time (white squares). The fluoroscopy sequence of the aluminum phantom was used

The Poissonian noise model considered by the NVCA algorithm supposes spatially and temporally adjacent pixels as uncorrelated. To test this hypothesis, the average spatial and temporal autocorrelation functions were evaluated from the fluoroscopy sequence of the aluminum step phantom (see the “Noise characterization”) and reported in Fig. 1. As expected, the correlation computed over time resulted very little (i.e. for a single inter-frame time interval scored 0.09 a.u.^2^). Also the correlation computed over space resulted limited (i.e. for a single pixel size scored 0.15 a.u.^2^).

**Fig. 2:**
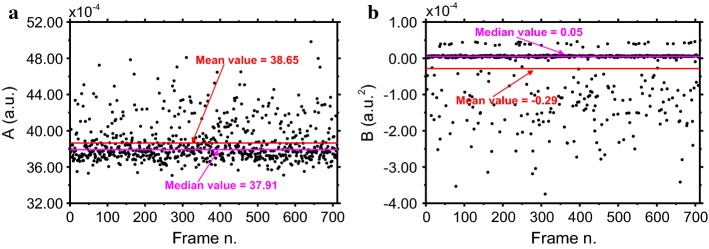
**a** *A* and **b** *B* coefficients estimated via the algorithm provided in ref [33]. Since *A* and *B* are estimated for each frame, the image frame number is reported on the *x*-axis

**Fig. 3:**
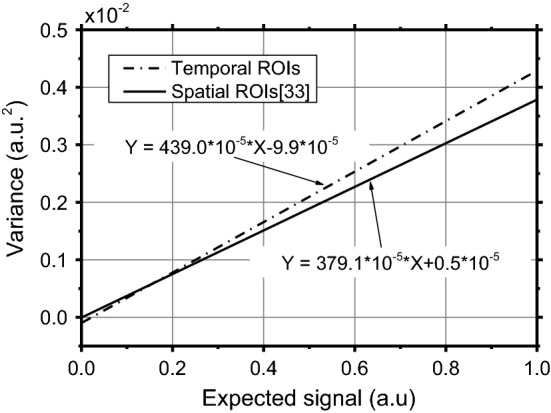
Expected value–variance relationship as estimated in space according to Ref. [33] and in time using the fluoroscopy sequence of the aluminum phantom

Since the NVCA algorithm and the Anscombe transform both require the characterization of the noise parameters (see “Noise characterization” section) they were estimated according to Ref. [33] (Fig. 2). They were evaluated for each of the 712 images of the aluminum phantom. The median *A* and *B* values resulted 37.91 × 10^−4^ a.u. (std dev = 2.25 × 10^−4^) and 0.05 × 10^−4^ a.u.^2^ (std dev = 0.75 × 10^−4^). The expected value–variance relationship evaluated using median values from Fig. 2 and that evaluated from the temporal sequence of the aluminum phantom are reported in Fig. 3. In the latter, the *A* and *B* parameters resulted 43.90 × 10^−4^ a.u. and − 0.99 × 10^−4^ a.u.^2^, respectively, and the *R*^2^ fit coefficient of the linear regression scored a value of 0.9196.

### Image quality evaluation (static scene)

**Fig. 4:**
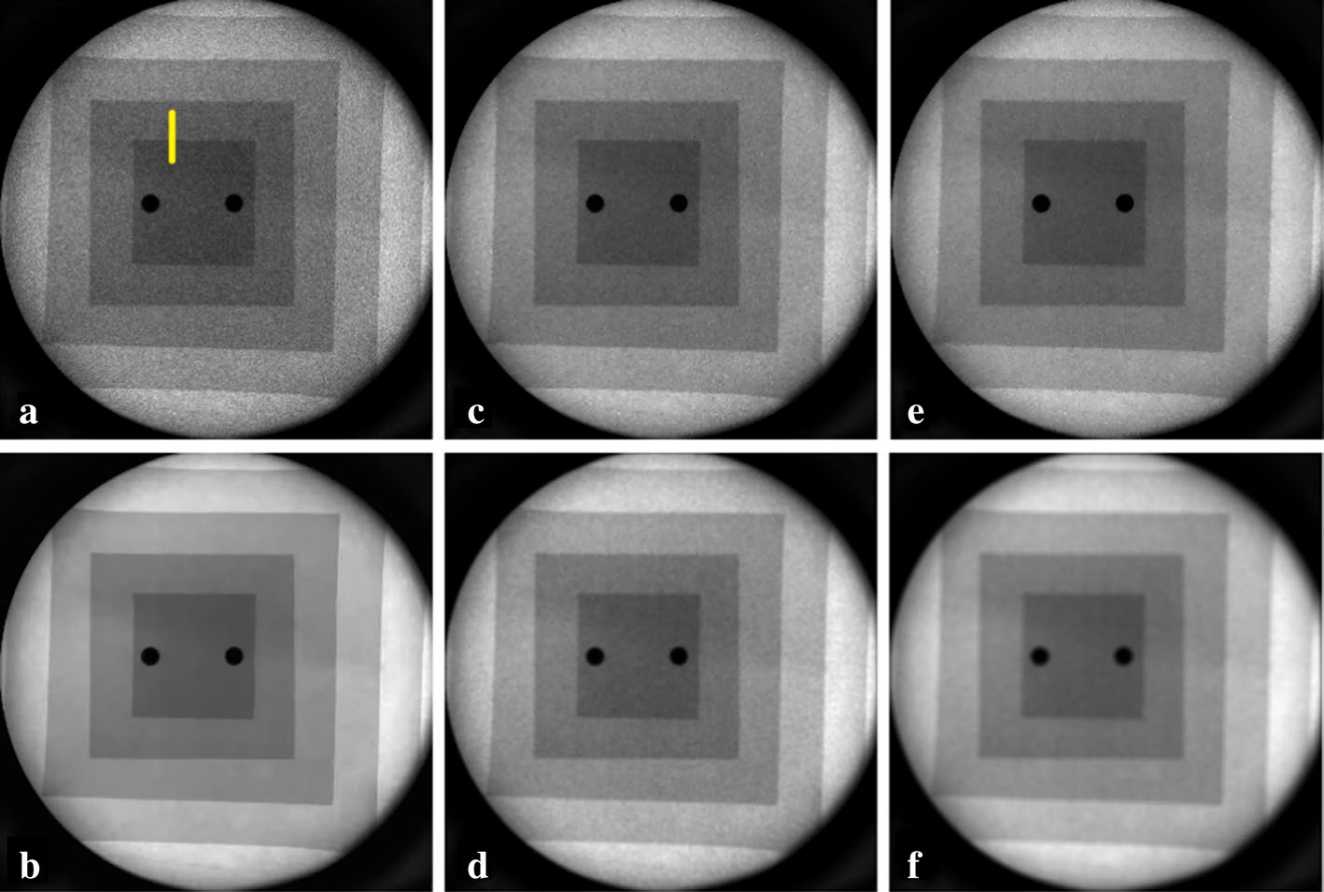
**a** Raw image; **b** VBM4D with algebraic inversion; **c** NVCA, 3 × 3 × 3 mask *T* = 2*σ*; **d** moving average 3 × 3 × 3 mask; **e** NVCA, 5 × 5 × 5 mask *T* = 2*σ*; **f** moving average 5 × 5 × 5 mask. The yellow vertical line in **a** indicates the profile selected for the spatial resolution evaluation

**Fig. 5:**
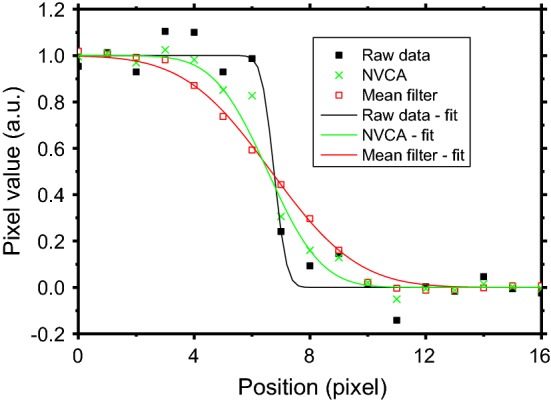
Profiles across the edge outlined in Fig. 4a for the raw image, the image filtered via NVCA algorithm (7 × 7 × 7 mask *T* = 2*σ*) and that filtered with a 7 × 7 × 7 average filter. Continuous lines represent the fitting curves adopted. The fitting parameter *R*^2^ is 0.9782 for the raw data, 0.9970 in the case of the average filter and 0.9977 in the case of the NVCA filter

Figure 4 shows a fluoroscopic frame of the aluminum phantom sequence without any filtering (Fig. 4a) and after the application of VBM4D algorithm (Fig. 4b), NVCA filter using a 3 × 3 × 3 pixels mask (Fig. 4c) and a 5 × 5 × 5 pixels mask (Fig. 4e) with a discrimination threshold of 2*σ*, moving average filter using a 3 × 3 × 3 pixels mask (Fig. 4d) and a 5 × 5 × 5 pixels mask (Fig. 4f). Figure 5 reports the profile across the edge outlined in yellow in Fig. 4a for the raw data and for the data processed with the moving average filter and the NVCA filters both using a 7 × 7 × 7 pixels mask size. The adopted fitting curves (see Eq. 3 in “Image quality parameters” section) achieved *R*^2^ fitting parameters higher than 0.9782.

**Fig. 6:**
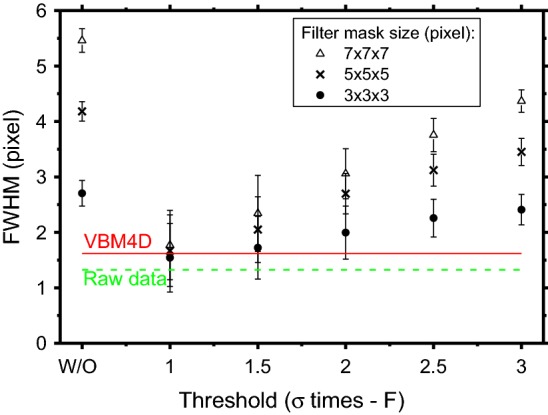
FWHM values for the evaluated filters and the raw data. The W/O label corresponds to the moving average filters

For the NCVA filter, the spatial resolution deteriorated as the threshold level T increased (Fig. 6). Indeed, the evaluated FWHM for 7 × 7 × 7 pixels mask presented a value of 1.8 pixels for a threshold equal to 1*σ*, and it increased up to 3.1 pixels and 4.4 pixels for threshold values of 2*σ* and 3*σ*, respectively. For a given threshold level, the larger the filter size the larger FWHM values. All the NVCA filters considered in Fig. 6 led to better spatial resolutions with respect to those obtained by conventional moving average filters with the same mask size. For instance, this produced a line spread function FWHM of 5.5 pixel for a 7 × 7 × 7 pixels mask, 77% larger than NVCA with the same mask size and 2*σ* threshold level. The NVCA filter with the threshold equal to 1*σ* provided a FWHM comparable to that produced by the VBM4D. For larger threshold levels, NVCA produced worse spatial resolutions than that achieved by VBM4D (e.g., for a threshold equal to 2*σ* the FWHM resulted 24%, 67% and 89% larger than VBM4D for 3 × 3×3, 5 × 5 × 5 and 7 × 7 × 7 pixel masks, respectively).

**Fig. 7:**
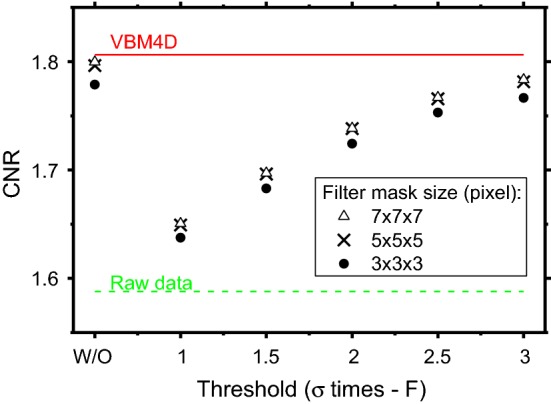
CNR values for the NVCA filters, VBM4D and raw data. The W/O label corresponds to the moving average filters

Although an increase of the NVCA mask size from 3 × 3 × 3 pixels to 5 × 5 × 5 pixels produced a slight improvement in CNR values (see Fig. 7), a further increase in the NVCA mask dimension did not produce further CNR improvements. The image CNR strongly depended on the NVCA threshold level: considering a 5 × 5 × 5 pixels mask, the CNR resulted equal to 1.65 for a threshold equal to 1*σ* and 1.78 for 3*σ*. The NVCA algorithm (5 × 5 × 5 pixels mask size and a threshold of 2*σ*) increased the CNR of 10%, while the VBM4D and the moving average filter (5 × 5 × 5 pixels mask) led to 14% and 13% CNR increase, respectively, when compared to the raw images.

**Fig. 8:**
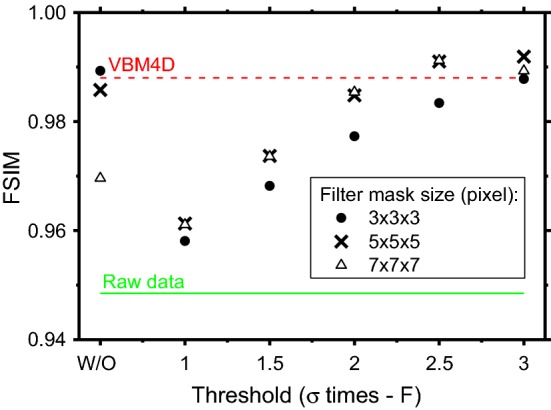
FSIM indices obtained using the NVCA, VBM4D and the average filter (labeled W/O) denoisers. It was evaluated on the static image of the aluminum step phantom; the reference image was obtained by averaging over time all the raw fluoroscopic frames

Figure 8 reports the FSIM global quality index computed for the NVCA-filtered images considering different mask sizes and different thresholds. The figure also includes the result obtained for the moving average filter (labeled as W/O on the *x*-axis). For threshold levels of 2.5*σ* and 3*σ*, the NVCA led to FSIM values comparable or larger than those of the VBM4D.

### Image quality evaluation (moving object)

**Fig. 9:**
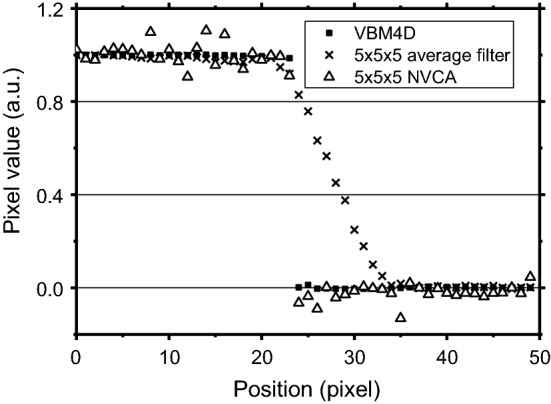
Edge profiles across the edge of the moving object in the sequence of the digital phantom for 5 × 5 × 5 average filter, VBM4D and 5 × 5 × 5 NVCA filter (*T* = 2*σ*). Insert speed = 1 pixel/frame

**Fig. 10:**
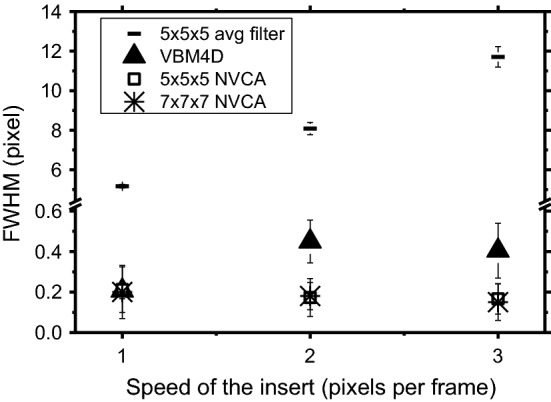
FWHM evaluated across a moving edge in the digital phantom for 5 × 5 × 5 average filter, VBM4D and 5 × 5 × 5 and 7 × 7 × 7 NVCA filter (*T* = 2*σ*)

Figure 9 shows profile across the ideal edge of the moving object in the digital phantom sequence (see “Sequences with moving objects” section) after the application of VBM4D, NVCA and moving average filter with 5 × 5 × 5 pixels mask size. It can be noted that the simple average filter introduced a conspicuous motion blur. On the contrary, both VBM4D and NVCA well preserved the edge sharpness (VBM4D provided a higher noise reduction). FWHM evaluated across this edge is reported in Fig. 10. For the moving object speed of 1 pixel/frame, differences in motion blur introduced by VBM4D and NVCA resulted negligible. However, the VBM4D spatial resolution degraded as the object speed increased, while the NVCA spatial resolution remained unaltered. In the first case, the FWHM increased of 2.2 times by increasing the insert speed from 1 to 2 pixels/frame. The moving average filter led to FWHM values more than 20 times higher than those of the other filters; the FWHM increased as the object speed increased.

**Fig. 11:**
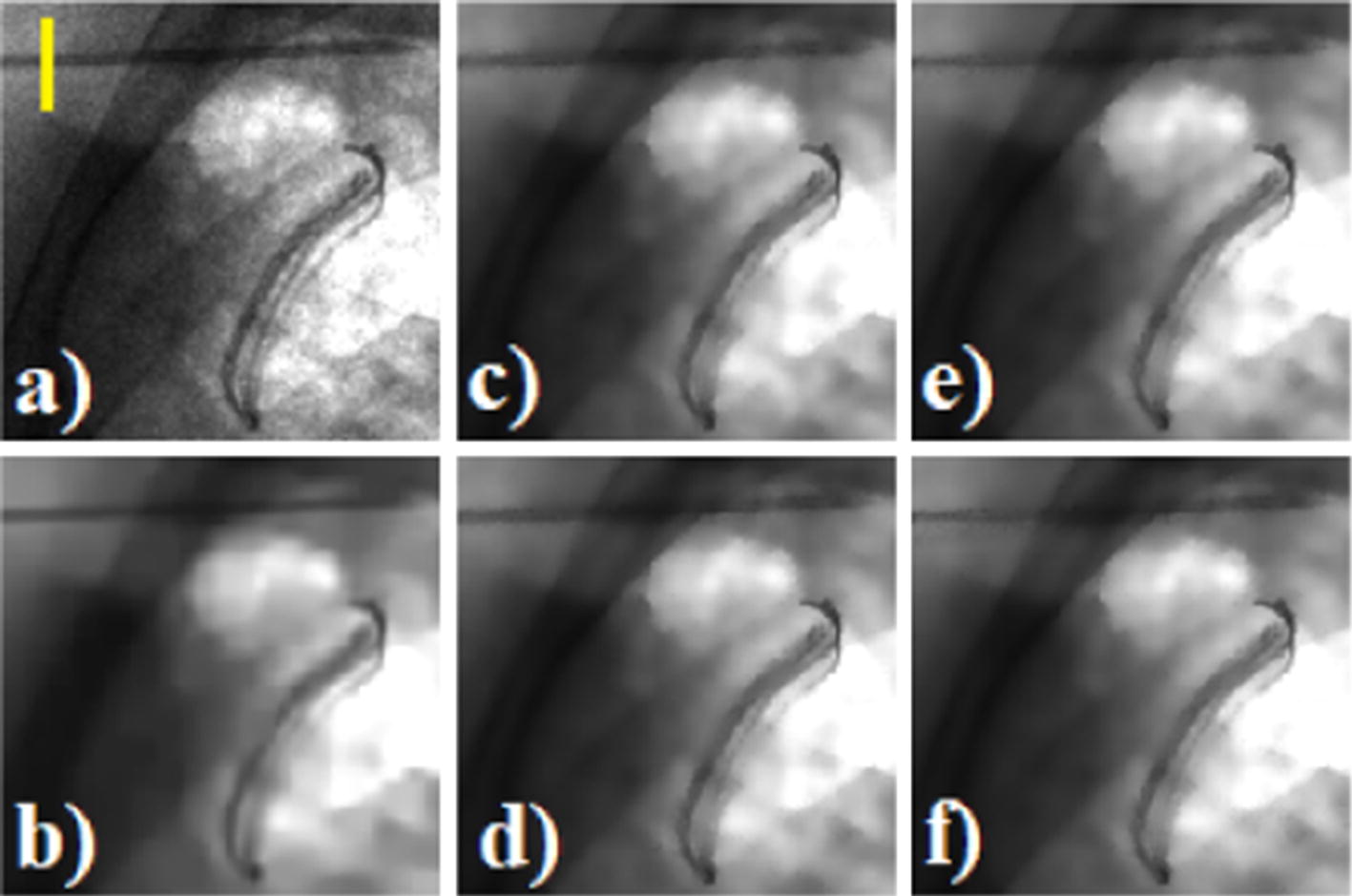
The enlargement of the real fluoroscopic image including the radiopaque needle: **a** raw image, the yellow vertical line was manually placed across the needle to evaluate blur, **b** image filtered with VBM4D and algebraic inversion of the Anscombe transform; image filtered with NVCA (*T* = 1.5*σ*) with mask size of **b** 5 × 5 × 1 pixels, **d** 5 × 5 × 3 pixels, **e** 5 × 5 × 5 pixels and **f** 5 × 5 × 7 pixels

**Fig. 12:**
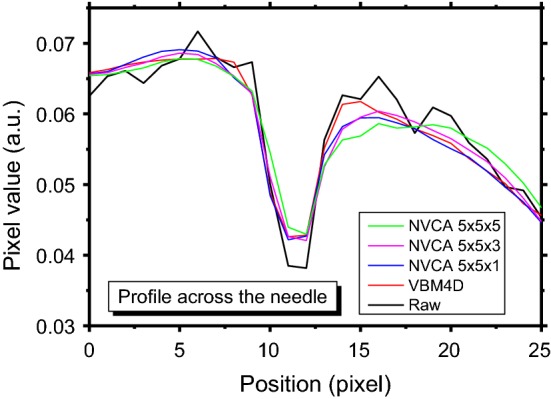
Profile across the needle outlined in Fig. 11a

Non-isotropic NVCA mask sizes were also tested. Figure 11 shows a region of interest (ROI) of the surgical sequence without any filtering and after the application of VBM4D and NVCA filter (threshold = 1.5*σ*) with mask size of 5 × 5 × 1 pixels (spatial filter), 5 × 5 × 3 pixels, 5 × 5 × 5 pixels and 5 × 5 × 7 pixels. No substantial differences in the profile blurring are caused by passing from a pure spatial filter (5 × 5 × 1 pixels mask) to a mask size of 5 × 5 × 3 pixels (Fig. 12). However, increasing the mask to 5 × 5 × 5 pixels led to a reduction in the signal peak level along with a profile spread (Fig. 12).

To better appreciate the differences in image quality obtained with the compared algorithms, we added in Additional files two demonstration videos showing raw and filtered sequences simultaneously. The videos were obtained from the surgical sequence (Additional file 1) and from the aluminum step phantom sequence (Additional file 2).

### Relative computational time

In Table 1, the computational times of the moving average and the NVCA filters are reported as percentages of the corresponding computational time of the VBM4D. As expected, computational times for NVCA filters resulted just few percent of that required for the VBM4D. Hardware implementations (such as [22, 23]) can further reduce the computational time using a lookup table instead of calculating the noise-threshold pixel by pixel.

**Table 1:** Percent relative computational times of the NVCA and moving average (MA) filters calculated with respect to the computational time of VBM4D, taken as reference

Spatial mask dimension	3 × 3						5 × 5						7 × 7					
Temporal mask dimension	1	2	3	5	7	9	1	2	3	5	7	9	1	2	3	5	7	9
NVCA-to-VBM4D computation time (%)	1.5	2.1	2.4	3.1	3.7	4.3	2.7	3.9	4.7	6.6	8.5	10.0	4.7	6.4	8.1	11.7	15.2	18.3
MA-to-VBM4D computation time (%)	1.1	1.2	1.2	1.3	1.4	1.5	1.1	1.4	1.7	2.1	2.3	2.7	1.6	2.0	2.5	3.1	3.7	4.3

## Discussion

Nowadays, patient’s dose during fluoroscopic procedures remains high, with peak skin doses up to several hundreds of mGy [34, 35]. This relatively high X-ray dose is mainly due to the long and unpredictable duration of the medical procedures and to the need to achieve an acceptable image quality (the SNR increases using higher doses). Vendors promote new equipment to reduce patient’s dose, some of which showing promising preliminary results [36–38]. Although the technology of X-ray detectors is improving [38], the quantum noise remains a physical limit and it can be only reduced by means of image processing methods. Furthermore, such processing must be achieved in real time to support clinical procedures. In many commercial fluoroscopic devices, only simple temporal (as low frame rate protocols) or simple spatial average filters are adopted. However, simple averaging compromises image sharpness, smears edges and produces motion blur. Several authors propose methods which couple filtering processes with edge detection techniques [19, 39, 40]. Although these approaches permit relative good results in terms of noise reduction and edge preservation, the implementation algorithms are still too complex and could compromise the real-time computation for on-line implementation during fluoroscopic exams. The proposed NVCA filter overcomes these limits, operating both in time and space while keeping a low computational complexity to allow real-time video processing and hardware implementation. The filter needs a preliminary estimation of the expected value–variance relationship of the noise (i.e., knowledge of the mixed Poissonian–Gaussian noise level). Only the surrounding pixels (in space and time) whose contrast is below the local noise intensity actually contribute to denoising, thus preserving edges (also of moving objects). The influence of the NVCA mask dimension and the noise-threshold on the image spatial resolution (line spread function FWHM) and on the CNR was tested. The algorithm proved capable to reduce noise (CNR resulted increased) and, at the same time, not to smooth out image edges. As in recent studies on low-dose X-ray-based imaging denoising [32, 40, 41], the NVCA performances were tested against the current state-of-the-art block-matching four-dimensional VBM4D video denoising. The noise variance of the fluoroscopic sequences considered for the comparison was stabilized via the Anscombe transformation to successfully apply the VBM4D.

## Conclusions

We presented and evaluated the performance of NVCA algorithm in terms of details visibility and spatial resolution of the filtered fluoroscopic sequences. We compared the proposed algorithm to the spatio-temporal moving average filter and to the VBM4D, assumed as gold standard. The NVCA algorithm demonstrated to preserve edge sharpness better than simple moving average filter. For a mask size of 7 × 7 × 7 pixels, the moving average filter produced a line spread function with FWHM 77% larger than that produced via NVCA with same mask size (2*σ* threshold). For low-threshold levels, the spatial resolution obtained with NVCA filter resulted comparable to that for VBM4D. In addition, the edge sharpness preservation resulted not to depend on the speed of the objects in the case of NVCA; this feature was not present in the images processed via VBM4D algorithm and moving average filter. For a mask size of 5 × 5 × 5 pixels and a noise-threshold level equal to 2 times the estimated noise standard deviation, the proposed filter produced a 10% increase of the CNR, while the much more complex VBM4D algorithm produced a 14% increase. The image global quality index FSIM showed that performance of NVCA for large threshold levels is comparable to that of VBM4D. The computational times required for NVCA filtering resulted just few percent of that required by VBM4D, confirming the theoretical predictions on their computational complexities, and verifying the possibility to perform real-time processing. In future, a hardware implementation of NVCA could be embedded in real fluoroscopic devices and the achieved SNR increase could be used to further reduce the patient’s dose during clinical procedures. However, since the mask size and noise-threshold influenced the final image quality, more extensive studies are required to assess the best settings. It is also important to specify that, obviously, NVCA denoising tends to make objects or details disappear if their contrast level is below the noise level.

## Methods

### Noise characterization

In a fluoroscopy system, the photons emerging from a patient and detected by the sensor can be described as a temporally stochastic Poisson process [9–11, 17]. However, a Poisson random variable with a relatively large average (i.e., larger than 10) can be locally well approximated by a Gaussian distribution with variance equal to the average [42]. If more than 10 photons are detected in the pixel area (this hypothesis is largely satisfied in actual fluoroscopic applications), the Gaussian approximation leads to a relative error lower than 0.1%, which becomes even smaller as the number of detected photons increases [42]. Since noise variance depends on the average pixel gray level (related to average number of detected photons), it is not constant over the whole image (heteroscedasticity), and the noise results to be signal dependent. Therefore, the actual gray level of any pixel in the image can be now decomposed as the sum of a noise-free signal and an additive, zero-mean, signal-dependent Gaussian noise, as expressed in Eq. 1:

1 $$g\left( {x,y} \right) = h\left( {x,y} \right) + {\mathcal{N}}\left( {0,\sigma^{2} \left( {h\left( {x,y} \right)} \right)} \right),$$

where (*x*,*y*) are the coordinates of the considered pixel on the detector plane, $g\left( {x,y} \right)$ is the actual pixel value, $h\left( {x,y} \right)$ is the noise-free signal and ${\mathcal{N}}\left( {0,\sigma^{2} \left( {h\left( {x,y} \right)} \right)} \right)$ is a Gaussian variable with zero mean and variance $\sigma^{2} \left( {h\left( {x,y} \right)} \right)$ which is a function of the expected value of the actual pixel luminance.

For a pure Poisson random variable (i.e., the ideal photon counting process on a single pixel), $\sigma^{2} \left( {h\left( {x,y} \right)} \right)$ is equal to $h\left( {x,y} \right)$. However, in practice, the detector gain and the detector noise modify this relationship. The actual value of the image pixel can be rather described as a random variable which is proportional to the photon count by the detector gain plus the detector noise background [10], resulting in a Poissonian–Gaussian mixture [33]. In this case, the expected value–variance relationship is a straight line, with a slope depending on the detector gain plus a constant due to detector noise and can be described as:

2 $$\sigma \left( {h\left( {x,y} \right)} \right)^{2} = A \cdot h\left( {x,y} \right) + B,$$

where *A* and *B* are constant values, referred to as noise parameters [33]. The proportionality coefficient (*A*) is determined by the detector gain, while the offset coefficient (*B*) is determined by the additive, signal-independent Gaussian noise deriving from the detector itself and electronics.

**Fig. 13:**
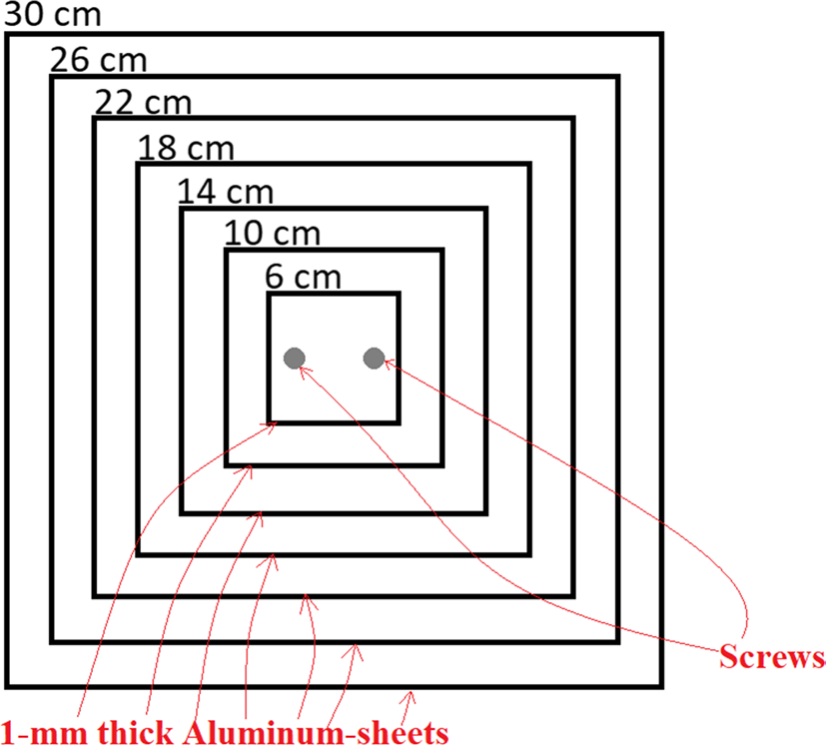
Schematic of the home-made test object used for the noise characterization

For noise characterization, a home-made phantom—whose scheme is depicted in Fig. 13—was used. It is composed of seven superimposed square aluminum sheets with edges of 30 cm, 26 cm, 22 cm, 18 cm, 14 cm, 10 cm and 6 cm. Each aluminum sheet was 1-mm thick. They were piled up with the centers placed on the same axis to produce a step phantom whose projected images presented flat regions with different average values. The sheets were fixed together by two metallic screws and bolts (visible in the object image as two black round shapes).

Phantom images were acquired with a GE 9900 Elite C-arm [43] fluoroscopic device. The X-ray tube was manually set to 57 kVp and 1 mA to simulate a real cardiac interventional procedure. The round field of view at the patient table had a diameter of 9 inch, the fame rate was set to 25 fps and the gray values were digitized in a 16-bit scale. The phantom image sequence was composed of 712 consecutive frames of 328 × 333 pixels.

The expected value–variance curve was estimated using the method presented in refs. [33, 44, 45] by means of the Matlab routine provided by the proposers [46]. The routine provides an automatic image segmentation of uniform regions and estimates the *A* and *B* coefficients by taking also into account the data clipping.

**Fig. 14:**
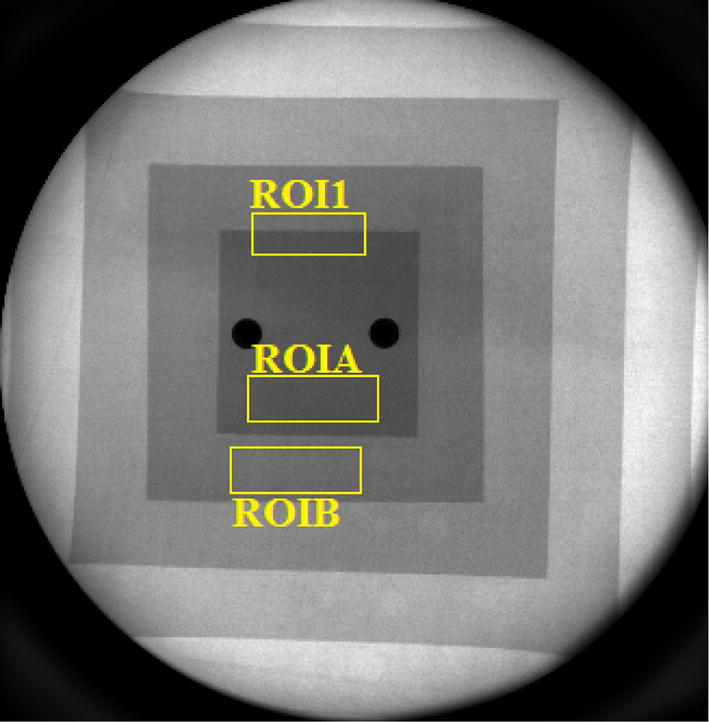
A frame of the aluminum step phantom fluoroscopic sequence. The manually selected regions of interest are shown in yellow: the ROI1 was chosen for the FWHM estimation and the ROIA and ROIB were chosen for CNR estimation. The two dark circles in the middle correspond to the two metallic screws and bolts that hold the aluminum sheets together

The Poissonian noise model described in the previous section assumes that image pixel values are uncorrelated. However, the limited spatial resolution of the imaging system determines correlation between adjacent pixels in the spatial domain. To quantify such a correlation, the 2D autocorrelation matrix of the ROI labeled as ROIA in Fig. 14 was computed. This ROI was selected on a flat, uniform region (i.e., not including edges) at the center of the field of view. The 2D autocorrelation matrix of ROIA was calculated for each frame of the image sequence and the average value was considered. Finally, the 1D autocorrelation curve was calculated as the radial profile of the 2D matrix.

However, actual fluoroscopic devices present time responses much shorter than the sampling times (few ms vs. more than 33 ms) and image pixels show weak correlation in the temporal domain. Therefore, considering a motionless test object and a given image pixel, the temporal sequence of the pixel values are independent samples of a Poisson random variable. For each image pixel, the local expected value and variance can be estimated considering the time sequence of the pixel values and the expected value–variance relationship can be obtained via linear regression. The obtained relationship was also compared to those obtained with the methods described in refs [33, 44, 45]. The temporal correlation of consecutive pixel values was also studied by computing the 1D autocorrelation of the time sequence of each image pixel and averaging over all pixels.

### Noise reduction algorithms

The NVCA algorithm has been already presented in previous papers [17, 21]. It is a conditioned spatio-temporal average filter that excludes from the average computation all those pixels values presumably not belonging to the local noise statistics. The 2D images sequence is seen as a 3D matrix. For a single pixel, the filter considers the nearby pixels which are comprised in *N* × *N* × *K* mask, where *K* is the temporal extent of the filter and *N* the spatial extent (usually *N* is an odd number to guarantee symmetry with respect to the central pixel). The value of the generic filtered pixel *I*_f_(*x*,*y*,*t*) is computed according to the formula:

3 $$I_{\text{f}} \left( {x,y,t} \right) = \frac{{\mathop \sum \nolimits_{i = 0}^{K - 1} \mathop \sum \nolimits_{{j = - \left( {N - 1} \right)/2}}^{{ + \left( {N - 1} \right)/2}} \mathop \sum \nolimits_{{h = - \left( {N - 1} \right)/2}}^{{ + \left( {N - 1} \right)/2}} \left( {C_{ijh} \cdot I\left( {x - h,y - j,t - i} \right)} \right)}}{{\mathop \sum \nolimits_{i = 0}^{K - 1} \mathop \sum \nolimits_{{j = - \left( {N - 1} \right)/2}}^{{ + \left( {N - 1} \right)/2}} \mathop \sum \nolimits_{{h = - \left( {N - 1} \right)/2}}^{{ + \left( {N - 1} \right)/2}} C_{ijh} }},$$

where *x* and *y* are the spatial coordinates and t the temporal coordinate; *I*(*x*, *y*, *t*) represents the unfiltered pixel value; *h* and *j* indices represent pixels spatial displacement with respect to the central pixel (corresponding to (*h*, *j*) = (0, 0)) and the *i* index represents the previous frame number with respect to current frame (corresponding to *i* = 0). The *C*_*ijh*_ coefficient assumes the value 0 or 1 according to the following decision rule:

$$\left\{ {\begin{array}{*{20}c} {C_{ijh} = 0 \quad if \left| {I\left( {x - h,y - j, t - i} \right) - I\left( {x,y,t} \right)} \right| > T\left( {I\left( {x,y,t} \right)} \right)} \\ {C_{ijh} = 1 \quad if \left| {I\left( {x - h,y - j, t - i} \right) - I\left( {x,y,t} \right)} \right| \le T\left( {I\left( {x,y,t} \right)} \right)} \\ \end{array} } \right.,$$

where the threshold value *T* is assumed equal to F times the local noise standard deviation (i.e., *T* = *F* × *σ*(*I*(*x*, *y*, *t*))). In other words, an adjacent pixel is included in the average computation only if its value is supposed to belong to the noise distribution associated to the central pixel. This approach helps to preserve the object edges both in space and over time. In the hardware implementation of the NVCA filter [22, 23], the predetermined T thresholds corresponding to the entire range of the image gray levels were stored into a lookup table to guarantee a faster computation.

The NVCA Matlab source code is available as Additional file 3: NVCN.m. This Matlab (MathWork Inc, version R2016b) function produces the NVCA-filtered sequence, requiring as inputs the raw fluoroscopy sequence, the spatial (*N*) and temporal (*K*) filter dimensions, the noise coefficients (*A* and *B*) and the *F* value. It is worth noting that, differently from the hardware implementation, the Matlab routine does not use a lookup table, but rather calculates the *T* value from the *F*, *A* and *B* parameters for each pixel.

In this work, the NVCA algorithm was compared to spatial–temporal moving average filters and the VBM4D algorithm. The generalized Anscombe transformation [15] was used to stabilize the Poissonian–Gaussian variance before the VBM4D filtering; after, the inverse algebraic Anscombe transform was applied [15].

In VBM4D, spatio-temporal volumes are constructed by tracking blocks along trajectories defined by the motion vectors and mutually similar volumes are stacked along an additional fourth dimension, thus producing a 4D structure, termed group [24, 25]. The VBM4D is an evolution of the block matching-3D (BM3D) [47] algorithm, originally designed for single image denoising, as it extends the paradigm of non-local grouping and collaborative filtering to exploit not only the spatial redundancy, but also the temporal redundancy characterizing video sequences. VBM4D filter needs to process the entire image sequence and it is very time consuming, therefore it does not allow real-time denoising. On the other hand, the block-matching processes permit to track and reallocate moving objects for a deep noise reduction and edge preservation. Because of these advantages VBM4D is a reference, state-of-the-art video denoiser [19, 23, 30–32, 39, 48]. The average filters (as the common fluoroscopy time-average pre-processor) allow fast and effective quantum noise suppression but they causes severe edge degradation and motion blur. NVCA is basically a simple advancement of the average filters, able to preserve edges even for moving objects and suitable for real-time denoising and hardware implementation. However, it requires a pre-estimation of the fluoroscopy noise and it is not suitable for retrieving details whose contrast is below the noise level.

### Image quality parameters

To evaluate the blurring introduced by the investigated filters, we estimated the FWHM of the line spread function. This last was evaluated as the derivative of the edge profile (edge spread function) across a manually selected sharp edge. To reduce the influence of the signal fluctuations on the FWHM estimates, we fitted the edge profile with the following function:

4 $$\psi \left( x \right) = 0.5 \times \left( {1 - {\text{erf}}\left( {\frac{x - c}{\sqrt 2 d}} \right)} \right),$$

where erf(·) indicates the error functions, *x* is a spatial variable (pixel) and *c* and *d* are fitting coefficients. The FWHM was evaluated from the fitting parameter *d* in the Eq. 4 as FWHM = 2.355 × *d*. It was estimated as the mean value over consecutive edge profiles and its uncertainty was estimated as the standard deviation over these estimates. For FWHM estimates in the case of fixed object, we considered 40 consecutive vertical profiles sampled in the ROI1 in Fig. 14, which shows an image of the aluminum step phantom.

To evaluate the increase in detail visibility, the CNR was computed as:

5 $${\text{CNR}} = \sqrt 2 \frac{{{\text{ROIA}}_{\text{mean}} - {\text{ROIB}}_{\text{mean}} }}{{\sqrt {{\text{ROIA}}_{\sigma }^{2} + {\text{ROIB}}_{\sigma }^{2} } }},$$

where ROIA_mean_ and ROIA_σ_ correspond to the average value and the standard deviation computed within the ROIA shown in Fig. 14 and ROIB_mean_ and ROIB_σ_ correspond to the average value and the standard deviation computed within the ROIB also shown in Fig. 14.

The FSIM [49] was used as a global index for image quality assessment. This index also considers the perception of the human eye, taking into account both the phase congruency and the gradient magnitude of an image. It was evaluated for a selected frame of the filtered versions of the sequence showed in Fig. 14, considering the time average of the whole sequence as the reference image.

### Sequences with moving objects

**Fig. 15:**
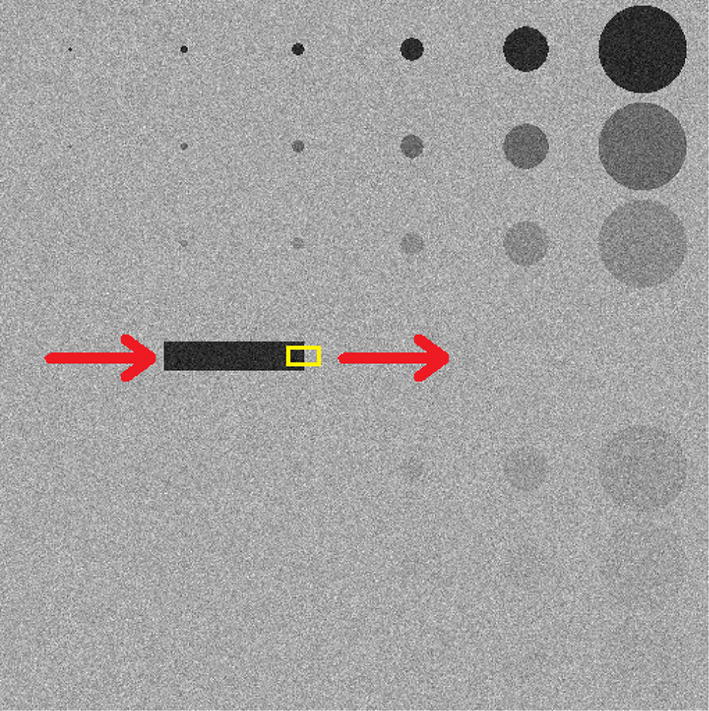
Digital phantom with a moving rectangular insert. The red arrows indicate the moving direction of the insert over the consecutive frames. The yellow ROI outlines the region for the evaluation of the FWHM on the insert edge

To evaluate the blurring introduced in the case of moving objects, an ad hoc, fully digital image sequence was created (Fig. 15). It included some motionless circular objects of different dimensions and contrasts, as well as a rectangular, high-contrast object moving horizontally from left to right (see red arrows in Fig. 15). The contrast between the moving object and the image background was 46% (evaluated as the percent ratio between the mean pixel value within the rectangle and the mean pixel value of the image background). The added Poissonian noise reflected that estimated in the experimental images. All the objects presented ideal edges. Three sequences were created with the mobile object moving at a speed of 1, 2 and 3 pixels per frame, respectively. The yellow ROI in Fig. 15 shows the edge portion selected for the FWHM evaluation (the ROI includes as much vertical edge as possible excluding the upper and lower corners). FWHM was estimated as the average value over 10 consecutive profiles. Its dimension was chosen to include as many lines as possible for reducing the noise influence on the FWHM estimates.

**Fig. 16:**
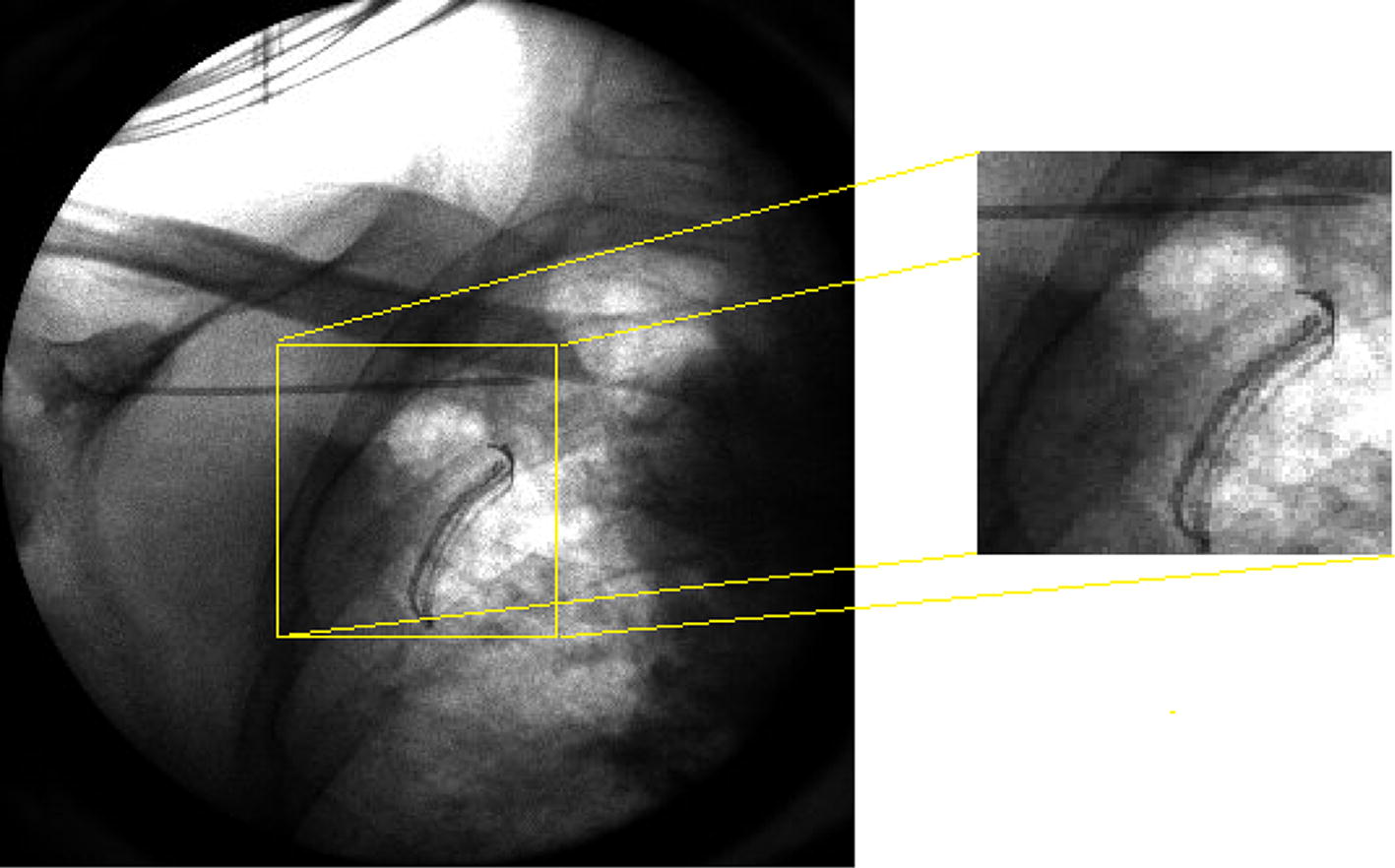
A fame of the surgical fluoroscopy sequence (left) and an enlargement of its central region (right). The radiopaque needle appears at the top of the image enlargement

In addition, the filters performances were evaluated using a real fluoroscopic sequence (Fig. 16) acquired during a surgical procedure. It shows a moving radiopaque needle. This sequence was acquired with the same device and settings used for the aluminum step phantom (GE 9900 Elite C-arm; tube voltage = 57 kVp, anode current = 1 mA; detector frame rate = 25 fps, frames dimension = 328 × 333 pixels and gray levels digitalized in a 16-bit scale).

### Computational complexity

The computational burden of the NVCA and VBM4D algorithms can be compared in terms of the number of required multiplications/divisions for an input of size *N*, which is generally expressed by the big-O notation [50, 51].

Let us consider a single frame of *N*-by-*N* pixels to be processed. The BM3D algorithm can be mainly decomposed in two phases: the block matching (grouping) and the 3D collaborative filtering. The block matching phase has a computational complexity order of *O*(*N*^4^*K*^2^), where *K* is the mask size [52, 53]. The 3D collaborative filtering phase provides for the processing of the 3D arrays identified by the block matching operation, and consists of 3D transformation, spectrum shrinkage, and 3D inverse transformation. Considering the case of a 3D-FFT transform [54] (which has a computational complexity of *O*(*N*·log *N*), with *N* being the total number of data to be processed) applied to a single 3D array composed by *S* similar blocks of K-by-K pixels, the computational complexity order can be expressed as *O*((*K*^2^*S*) log (*K*^2^*S*)).

Indicating with *R* and *G*, respectively, the total number of *K*-by-*K* blocks in the image, and the number of 3D arrays identified by the block matching operation, and assuming every single 3D array to be composed by *S* similar blocks, it is possible to express the computational complexity order of the whole 3D filtering operation as *O*(*G*·((*K*^2^*S*) log(*K*^2^*S*))). Since *G* = *R*/*S* and *R* = *N*^2^/*K*^2^, the computational complexity can be expressed as *O*(*N*^2^ log(*K*^2^*S*)).

If the block matching does not find any similar blocks (i.e., *S* = 1), the computational complexity order is *O*(*N*^2^ log *K*^2^). Alternatively, if the block matching provides a single 3D array composed by all the *R* blocks (i.e., *S* = *R*), the computational complexity order is *O*(*N*^2^ log *N*^2^). The computational complexity of the block matching is of some orders of magnitude higher than that of the 3D filtering, and so it dominates the computational complexity of the whole BM3D algorithm. Since VBM4D algorithm works along an additional dimension (i.e., time), thus processing a larger amount of data, its computational complexity is certainly higher than that of the BM3D.

The NVCA provides for a single division operation per pixel [17, 21–23], so for the same frame of *N*-by-*N* pixels, it has a computational complexity order of *O*(*N*^2^), which results several orders of magnitude lower than VBM4D’s one.

Actual computational times were estimated for moving average filter, NVCA and VBM4D filters to provide quantitative evidence. These three filters were applied to the 712 frames of the image sequence shown in Fig. 2 via MEX files (i.e., C/C++ subroutines created in Matlab—http://www.mathworks.com). They were run in Matlab R2016b on a PC equipped with an Intel Core i7-3770 CPU at 3.40 GHz (Windows 7 operating system). Relative computational times were evaluated as the ratio between the NVCA or moving average filtering time and that corresponding to the VBM4D.

## Supplementary information

**Additional file 1.** Demonstration videos showing raw and filtered sequences simultaneously of the surgical sequence. The frame rate was reduced 2.5 to 1.

**Additional file 2.** Demonstration videos showing raw and filtered sequences simultaneously of the aluminum step phantom sequence at 25 fps.

**Additional file 3.** The NVCA Matlab routine function. It produces the NVCA-filtered sequence, requiring as inputs the raw fluoroscopy sequence, the spatial (N) and temporal (K) filter dimensions, the noise coefficients (A and B) and the F value.

## Data Availability

All data generated or analyzed during this study are included in this published article.

## References

[1] Moradi M, Mahdavi SS, Dehghan E, Lobo JR, Deshmukh S, Morris WJ (2012). Seed localization in ultrasound and registration to C-Arm fluoroscopy using matched needle tracks for prostate brachytherapy. IEEE Trans Biomed Eng.

[2] Weese J, Penney GP, Desmedt P, Buzug TM, Hill DLG, Hawkes DJ (1997). Voxel-based 2-D/3-D registration of fluoroscopy images and CT scans for image-guided surgery. IEEE Trans Inf Technol Biomed.

[3] Bifulco P, Cesarelli M, Cerciello T, Romano M (2012). A continuous description of intervertebral motion by means of spline interpolation of kinematic data extracted by video fluoroscopy. J Biomech.

[4] Yamazaki T, Watanabe T, Nakajima Y, Sugamoto K, Tomita T, Yoshikawa H (2004). Improvement of depth position in 2-D/3-D registration of knee implants using single-plane fluoroscopy. IEEE Trans Med Imaging.

[5] Wang J, Zhu L, Xing L (2009). Noise reduction in low-dose X-ray fluoroscopy for image-guided radiation therapy. Int J Radiat Oncol Biol Phys.

[6] Cerciello T, Romano M, Bifulco P, Cesarelli M, Allen R (2011). Advanced template matching method for estimation of intervertebral kinematics of lumbar spine. Med Eng Phys.

[7] Ma L, Moisan L, Yu J, Zeng T (2013). A dictionary learning approach for Poisson image deblurring. IEEE Trans Med Imaging.

[8] Lefkimmiatis S, Maragos P, Papandreou G (2009). Bayesian inference on multiscale models for Poisson intensity estimation: applications to photon-limited image denoising. IEEE Trans Image Process.

[9] Tapiovaara MJ (1993). SNR and noise measurements for medical imaging: II. Application to fluoroscopic X-ray equipment. Phys Med Biol.

[10] Aufrichtig R, Wilson DL (1995). X-ray fluoroscopy spatio-temporal filtering with object detection. IEEE Trans Med Imaging.

[11] Wang J, Blackburn TJ (2000). The AAPM/RSNA physics tutorial for residents: X-ray image intensifiers for fluoroscopy. Radiographics.

[12] Lo CM, Sawchuk AA (1973). Nonlinear restoration of filtered images with Poisson noise.

[13] Gonzalez RC, Woods RE (1992). Digital image processing.

[14] Mäkitalo M, Foi A (2011). Optimal inversion of the Anscombe transformation in low-count Poisson image denoising. IEEE Trans Image Process.

[15] Mäkitalo M, Foi A (2013). Optimal inversion of the generalised Anscombe for Poisson-Gaussian noise. IEEE Trans Image Process.

[16] Bindilatti AA, Mascarenhas NDA (2013). A non local Poisson denoising algorithm based on stochastic distances. IEEE Signal Process Lett.

[17] Cesarelli M, Bifulco P, Cerciello T, Romano M, Paura L (2013). X-ray fluoroscopy noise modeling for filter design. Int J Comput Assist Radiol Surg.

[18] Lee MS, Park CH, Kang MG (2017). Edge enhancement algorithm for low-dose X-ray fluoroscopic imaging. Comput Meth Prog Biomed.

[19] Lee MS, Park SW, Lee SY, Kang MG (2017). Motion-adaptive 3D nonlocal means filter based on stochastic distance for low-dose X-ray fluoroscopy. Biomed Signal Process.

[20] Wagner M, Yang P, Schafer S, Strother C, Mistretta C (2015). Noise reduction for curve-linear structures in real time fluoroscopy applications using directional binary masks. Med Phys.

[21] Cerciello T, Bifulco P, Cesarelli M, Fratini A (2012). A comparison of denoising methods for X-ray fluoroscopic images. Biomed Signal Process Control.

[22] Genovese M, Bifulco P, De Caro D, Napoli E, Petra N, Romano M, Cesarelli M, Strollo AGM (2015). Hardware implementation of a spatio-temporal average filter for real-time denoising of fluoroscopic images. J VLSI.

[23] Castellano G, De Caro D, Esposito D, Bifulco P, Napoli E, Petra N, Andreozzi E, Cesarelli M, Strollo AGM (2019). An FPGA-oriented Algorithm for real-time filtering of poisson noise in video streams, with application to X-ray fluoroscopy. Circuits Syst Signal Process.

[24] Maggioni M, Boracchi G, Foi A, Egiazarian K. Video denoising using separable 4D nonlocal spatiotemporal transforms. In: Proc. SPIE electronic imaging 2011, image processing: algorithms and systems IX, 7870–2, San Francisco (CA), USA; 2011.

[25] Maggioni M, Boracchi G, Foi A, Egiazarian K (2012). Video denoising, deblocking and enhancement through separable 4-D nonlocal spatiotemporal transforms. IEEE Trans Image Proc..

[26] Burger HC, Schuler CJ and Harmeling S.Image denoising: Can plain neural networks compete with BM3D?,” In: 2012 IEEE conference on computer vision and pattern recognition. Providence, RI; 2012; p. 2392–2399.

[27] Nishio M, Nagashima C, Hirabayashi S, Ohnishi A, Sasaki K, Sagawa T, Hamada M, Yamashita T (2017). Convolutional auto-encoder for image denoising of ultra-low-dose CT. Heliyon..

[28] Anaya J, Barbu A (2018). RENOIR—a dataset for real low-light image noise reduction. J Vis Commun Image R.

[29] Hasan M, El-Sakka MJ (2018). Improved BM3D image denoising using SSIM-optimized Wiener filter. EURASIP J Image Video Process.

[30] Ehmann J, Chu L, Tsai S, Liang C. Real-time video denoising on mobile phones. In: 25th IEEE international conference on image processing (ICIP). Athens, 2018; p. 505–509.

[31] Furnival T, Leary RK, Midgley PA (2017). Denoising time-resolved microscopy image sequences with singular value thresholding. Ultramicroscopy.

[32] Shi L, Hu Y, Chen Y, Yin X, Shu H, Luo L, Coatrieux JL (2016). Improving low-dose cardiac CT images based on 3D sparse representation. Sci Rep.

[33] Foi A, Trimeche M, Katkovnik V, Egiazarian K (2008). Practical Poissonian–Gaussian noise modeling and fitting for single-image raw-data. IEEE Trans Image Process.

[34] Pasquino M, Cutaia C, Poli M, Valero C, Peroni G, De Benedictis M (2018). Patient’s Peak Skin Dose evaluation using Gafchromic films in interventional cardiology procedures and its correlation with other dose indicators. Phys Med.

[35] Greffier J, Moliner G, Pereira F, Cornillet L, Ledermann B, Schmutz L (2016). Assessment of patient’s peak skin dose using Gafchromic films during interventional cardiology procedures: routine experience feedback. Radiat Prot Dos.

[36] Spink C, Avanesov M, Schmidt T, Grass M, Schoen G, Adam G (2017). Noise reduction angiographic imaging technology reduces radiation dose during bronchial artery embolization. Eur J Radiol.

[37] Hoffmann R, Langenbrink L, Reimann D, Kastrati M, Becker M, Piatkowski M (2017). Image noise reduction technology allows significant reduction of radiation dosage in cardiac device implantation procedures. Pacing Clin Electroph.

[38] Plank F, Stowasser B, Till D, Schgör W, Dichtl W, Hintringer F (2019). Reduction of fluoroscopy dose for cardiac electrophysiology procedures: a feasibility and safety study. Eur J Rad.

[39] Amiot C, Girard C, Chanussot J, Pescatore J, Desvignes M (2016). Spatio-temporal multiscale denoising of fluoroscopic sequence. IEEE Trans Med Imaging.

[40] Hariharan SG, Strobel N, Kaethner C, Kowarschik M, Demirci S, Albarqouni S (2018). A photon recycling approach to the denoising of ultra-low dose X-ray sequences. Int J Comput Ass Rad.

[41] Zhao T, Hoffman J, McNitt-Gray M, Ruan D (2019). Ultra-low-dose CT image denoising using modified BM3D scheme tailored to data statistics. Med Phys.

[42] Hensel M, Pralow T, Grigat RR. Modeling and real-time estimation of signal-dependent noise in quantum-limited imaging. In: Proceedings of the 6th WSEAS international conference on signal processing, robotics and automation. Corfu Island, Greece, 2007; p. 183–191.

[43] http://www3.gehealthcare.ca/en/products/categories/surgical_imaging/oec_c-arms/oec_9900_elite. Accessed 15 July 2019.

[44] Foi A, Alenius S, Katkovnik V, Egiazarian K (2007). Noise measurement for raw-data of digital imaging sensors by automatic segmentation of non-uniform targets. IEEE Sens J.

[45] Azzari L, Foi A. Gaussian-Cauchy mixture modeling for robust signal-dependent noise estimation. In: Proc. 2014 IEEE Int. Conf. Acoustics, Speech, Signal Process. (ICASSP 2014) 2014; p. 5357–5361.

[46] http://www.cs.tut.fi/~foi/GCF-BM3D/. Accessed 15 July 2019.

[47] Dabov K, Foi A, Katkovnik V, Egiazarian K (2007). Image denoising by sparse 3D transform-domain collaborative filtering. IEEE Trans Image Process..

[48] Mildenhall B, Barron JT, Chen J, Sharlet D, Ng R, Carroll R. Burst denoising with kernel prediction networks. In: 2018 IEEE/CVF conference on computer vision and pattern recognition, Salt Lake City, UT, 2018; p. 2502–2510.

[49] Zhang L, Zhang L, Mou X, Zhang D (2011). FSIM: a feature similarity index for image quality assessment. IEEE Trans Image Proc.

[50] Bürgisser P, Clausen M, Shokrollahi MA (1997). Algebraic complexity theory.

[51] Atallah MJ (1999). Algorithms and theory of computation handbook.

[52] Brox T, Kleinschmidt O, Cremers D (2008). Efficient non-local means for denoising of textural patterns. IEEE Trans Image Proc.

[53] Dabov K, Foi A, Katkovnik V, Egiazarian K. Image denoising with block-matching and 3D filtering. In: Electronic Imaging’06, Proc. SPIE 6064, no. 6064A-30, San Jose, California USA, 2006.

[54] Foi A, Katvonik V, Egiazarian K (2007). Pointwise shape-adaptive DCT for high-quality denoising and deblocking of grayscale and color images. IEEE Trans Image Process.

